# Response to Huang et al. “Herpesviridae reactivation for poor outcome in ARDS patients with ECMO: criminal or witness?”

**DOI:** 10.1186/s13613-020-0627-3

**Published:** 2020-01-21

**Authors:** Sami Hraiech, Laurent Papazian

**Affiliations:** 1 Service de Médecine Intensive-Réanimation, APHM, Hôpital Nord, Marseille, France; 2 CEReSS-Center for Studies and Research on Health Services and Quality of Life EA3279, Aix-Marseille University, Marseille, France

Dear Mr Editor,

Huang et al. recently commented on our publication concerning *Herpesviridae* reactivation in patients under veno-venous extracorporeal membrane oxygenation (VV ECMO) for severe acute respiratory distress syndrome (ARDS) and raised some important questions. Data available do not allow to conclude once for all, however, we might provide some pieces of answers.

First, the “who?”. In a general population of ARDS patients (without ECMO), Ong et al. [1] showed that cytomegalovirus (CMV) reactivation among seropositive patients was diagnosed in 27% of patients and 34% among those with concurrent septic shock. In our cohort, CMV reactivation raised 40%, either alone or combined with Herpes Simplex virus (HSV), septic shock being present in nearly all reactivated patients at the time of intensive care unit (ICU) admission. It is not surprising that *Herpesviridae* reactivation is so common in ARDS patients under ECMO who often combine sepsis and prolonged mechanical ventilation (MV). It would be of high interest to perform a matched controlled study in order to determine if ECMO occurring during ARDS enhances the frequency of *Herpesviridae* reactivation.

Second, the “how?”. The precise definition of *Herpesviridae* reactivation or active infection in ICU patients is rather touchy. The positivity of biological samples signs the evidence of viral transition from latency to replication, but end-organ disease symptoms (i.e., pneumonia, hepatitis, colitis, hematological disorders, etc.) can be difficult to identify in such complex patients. One key question would be the comparison of performance diagnosis of blood and airway CMV PCR with antigenemia, which is considered as the reference test in end-organ disease diagnosis. Concerning the timing of HSV and CMV reactivation, Heininger et al. [2], comparing airway samples, already showed that HSV reactivation occured earlier during MV.

Finally, the “why?”. Several hypotheses have been stated to explain the poor outcomes of patients exhibiting *Herpesviridae* reactivation during ARDS [3]. Viral pneumonia due to direct lung aggression by HSV or CMV might be the first mechanism. This can be suggested when lung samples are positive but only characteristic histological lesions can affirm such a diagnosis, especially because lung imaging aspects are non-specific. The second mechanism might be an immunopathological effect, in which damage to tissues is caused by the excessive immune response to the virus with prolonged ARDS or evolution towards lung fibrosis. In our cohort, alveolar procollagen III was not different between both groups (24 vs 14% for reactivated and non-reactivated patients), but our study might be underpowered for this comparison and antiviral treatments might have interfered with this result. The third explanation is related to an alteration of patients’ immune defenses, making them more susceptible to fungal and bacterial infections. It would be of high interest to explore immune function in ARDS patients with *Herpesviridae* reactivation with specific markers such as monocytes HLA-DR expression [4].

To summarize, despite several decades of research, much remains to be done to lighten the role of *Herpesviridae* in ARDS patients, and even more in those under ECMO.

## Data Availability

Not applicable.

## Abbreviations

ARDS: acute respiratory distress syndrome
CMV: cytomegalovirus
HSV: Herpes Simplex Virus
MV: mechanical ventilation
VV ECMO: veno-venous extra corporeal membrane oxygenation
